# Antioxidant and Anti-Osteoclastogenic Potential of Olive Oil Dregs Extracts

**DOI:** 10.3390/molecules31183178

**Published:** 2026-09-10

**Authors:** Beatrice Castiglioni, Giuseppe Squillaci, Alice Marchetti, Marica Imbimbo, Adrian Perez-Barreto, Alessandra Morana, Virginia Carbone, Michela Bosetti

**Affiliations:** 1 Department of Pharmaceutical Sciences, University of Eastern Piedmont “A. Avogadro”, Largo Donegani 2, 28100 Novara, Italy; beatrice.castiglioni@uniupo.it (B.C.); alice.marchetti@uniupo.it (A.M.); marica.imbimbo@uniupo.it (M.I.); 2 Institute of Biochemistry and Cell Biology, National Research Council of Italy, Via Pietro Castellino 111, 80131 Naples, Italy; giuseppe.squillaci@cnr.it; 3 Blond McIndoe Laboratories, Division of Cell Matrix Biology and Regenerative Medicine, Faculty of Biology, Medicine, and Health, The University of Manchester, Oxford Road, Manchester M13 9PL, UK; adrian.perezbarreto@postgrad.manchester.ac.uk; 4 Research Institute on Terrestrial Ecosystems, National Research Council of Italy, Via Pietro Castellino 111, 80131 Naples, Italy; alessandra.morana@cnr.it; 5 Institute of Food Sciences, National Research Council of Italy, Via Roma 64, 83100 Avellino, Italy; virginia.carbone@cnr.it

**Keywords:** anti-osteoclastogenesis, antioxidant, bone loss, olive oil, waste, bioactive molecules, eco-friendly extraction

## Abstract

The agri-food industry generates substantial amounts of waste, which are increasingly regarded as valuable resources within the framework of the circular economy. Among the recoverable bioactive molecules, phenolic compounds are of particular interest for nutraceutical and biomedical applications. Olive oil production by-products, such as olive oil dregs (OOD), represent a rich source of polyphenols, especially hydroxytyrosol (3,4-DHPEA). In this study, we investigated the in vitro biological properties of OOD extracts obtained through methanolic or eco-friendly aqueous extraction methods, both characterized by a high 3,4-DHPEA content. The antioxidant potential of the extracts was evaluated together with their activity in murine peritoneal macrophages, while their ability to counteract osteoclastogenesis was assessed using RAW 264.7 cells stimulated with receptor activator of nuclear factor kappa-B ligand (RANKL). Our results demonstrated that both extracts showed a total antioxidant effect and significantly reduced intracellular reactive oxygen species (ROS) levels. Notably, this antioxidant activity was accompanied by a marked inhibition of RANKL-induced osteoclast formation, as evidenced by a reduction in tartrate-resistant acid phosphatase (TRAP)-positive multinucleated cells together with fewer mature osteoclasts displaying well-structured F-actin rings and bone-resorbing activity as demonstrated by the pit formation assay. Taken together, these findings suggest that OOD extract, through its dual antioxidant and anti-osteoclastogenic properties, may represent a promising strategy for the prevention of inflammation-driven pathological bone loss.

## 1. Introduction

The agri-food industry generates thousands of tons of by-products and residues each year, creating substantial challenges related to waste disposal and environmental contamination [1,2]. In line with the principles of the circular economy, these agro-industrial residues are no longer viewed as end-products, but as valuable sources of bioactive molecules that can be recovered and reintegrated into productive processes [3]. Among these, phenolic compounds (PCs) are particularly attractive due to their well-documented biological activities and potential applications in the nutraceutical and biomedical sectors [4].

Olive oil production, a historical milestone in Mediterranean culture, represents a paradigmatic example of this concept. While olive oil is widely recognized for its health-promoting effects, including cardioprotective, anti-inflammatory, and antioxidant properties, its extraction process generates large quantities of by-products, such as olive mill wastewater and olive oil filter cake. These residues retain a considerable amount of PCs and, therefore, represent potentially valuable sources of biologically active compounds. Beyond their antioxidant and anti-inflammatory effects, increasing evidence suggests that PCs can also influence bone remodeling by modulating the activity and differentiation of bone-resorbing osteoclasts. Although osteoclastogenesis is essential for physiological bone remodeling, excessive osteoclast formation and activity contribute to pathological bone loss.

Osteoclastogenesis is primarily driven by RANKL signaling, which activates downstream pathways including NF-κB, MAPK and NFATc1, with ROS acting as important intracellular mediators of this process [5,6]. Accordingly, several plant-derived polyphenols, including resveratrol, curcumin and quercetin, have been reported to suppress RANKL-induced osteoclast differentiation through modulation of oxidative stress and these downstream osteoclastogenic pathways [7,8,9]. Other natural compounds and plant-derived extracts have also been shown to influence RANKL/OPG signaling and reduce the expression of osteoclast-associated markers such as TRAP and cathepsin K [10]. Collectively, these findings highlight plant-derived phenolic compounds as promising candidates to limit excessive osteoclastogenesis and associated pathological bone resorption.

Among the phenolic compounds present in olive oil by-products, hydroxytyrosol (3,4-DHPEA) has attracted growing attention for its wide spectrum of biological effects [11]. Beyond its antioxidant and anti-inflammatory properties, accumulating evidence indicates a key role for 3,4-DHPEA in bone physiology and homeostasis. Experimental studies have demonstrated that 3,4-DHPEA exerts a marked inhibitory effect on osteoclastogenesis, thereby reducing the formation and activity of bone-resorbing cells. By attenuating inflammatory responses and limiting excessive osteoclast differentiation, this bioactive molecule helps to sustain a balanced bone remodeling process, thereby offering protection against inflammation-driven pathological bone loss [12,13]. These findings provide a strong rationale for investigating 3,4-DHPEA-rich olive-derived residues as potential sources of bioactive compounds capable of modulating osteoclastogenesis.

Despite this promising bioactivity, the large-scale use of 3,4-DHPEA is hindered by limitations in its availability and production. Chemical or enzymatic synthesis methods remain costly and labor-intensive [14,15]. In this context, the valorization of olive oil by-products as alternative, sustainable sources of 3,4-DHPEA and other PCs is gaining increasing attention. For instance, olive oil dregs (OOD), often considered waste material, represent underexplored reservoirs of bioactive phenolics, especially 3,4-DHPEA [16].

To our knowledge, the antioxidant and anti-inflammatory potential of OOD, based on different extraction methods, remains poorly characterized. Here, we assess its phenolic profile and its effects on intracellular ROS and RANKL-induced osteoclastogenesis.

## 2. Results

### 2.1. 3,4-DHPEA Quantification and Estimation of Total Antioxidant Capacity

As 3,4-DHPEA is considered one of the most powerful antioxidants among the phenolic compounds of the olive tree [17], the in vitro tests were conducted using the OOD extracts with reference to their 3,4-DHPEA content and compared to the 3,4-DHPEA standard (3,4-DHPEA Std.) at the same concentration. For this purpose, the 3,4-DHPEA amount was estimated in both acidified methanol and acidified water OOD extracts, and results are shown in Table 1.

**Figure 1:**
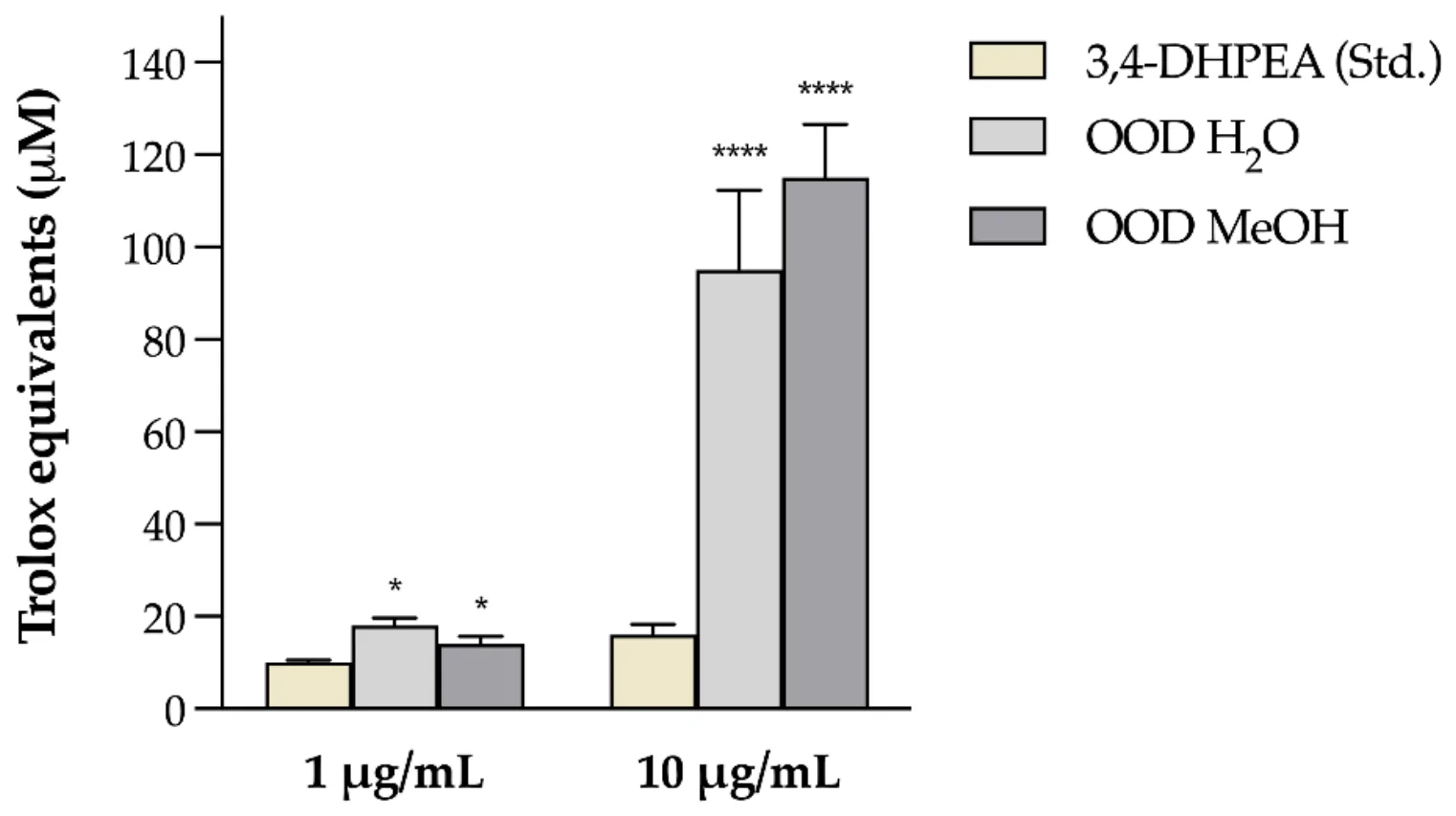
Total antioxidant capacity of OOD extracts. Methanolic and aqueous OOD extracts were tested at concentrations of 1 µg/mL and 10 µg/mL, with concentrations referring to the 3,4-DHPEA content in the extracts. Hydroxytyrosol standard (3,4-DHPEA Std.) was used as a reference. The Trolox equivalent antioxidant capacity (TEAC) is expressed as Trolox equivalents (µM). Data are presented as the mean ± SEM, based on three independent experiments performed in duplicate. Statistical significance was determined by one-way ANOVA followed by Tukey’s test (* *p* < 0.05, **** *p* < 0.0001), indicating a significant difference from the 3,4-DHPEA Std.

The total antioxidant capacity of both methanolic and aqueous OOD extracts at concentrations of 1 and 10 µg/mL was assessed (Figure 1). The results show that both extracts, regardless of the extraction method, exhibit a very high antioxidant capacity in a dose-dependent manner. When comparing the results obtained with the 3,4-DHPEA Std., the antioxidant activity showed a more pronounced difference at the 10 µg/mL concentration. These findings suggest that other bioactive compounds, although present in smaller quantities, may contribute to the enhanced antioxidant activity. While a potential synergistic antioxidant effect among olive oil polyphenols has been noted in the literature, the optimal ratio between them remains unclear and is an area of ongoing research [18].

### 2.2. OOD Extracts Decrease Intracellular ROS Levels in Murine Peritoneal Macrophages

**Figure 2:**
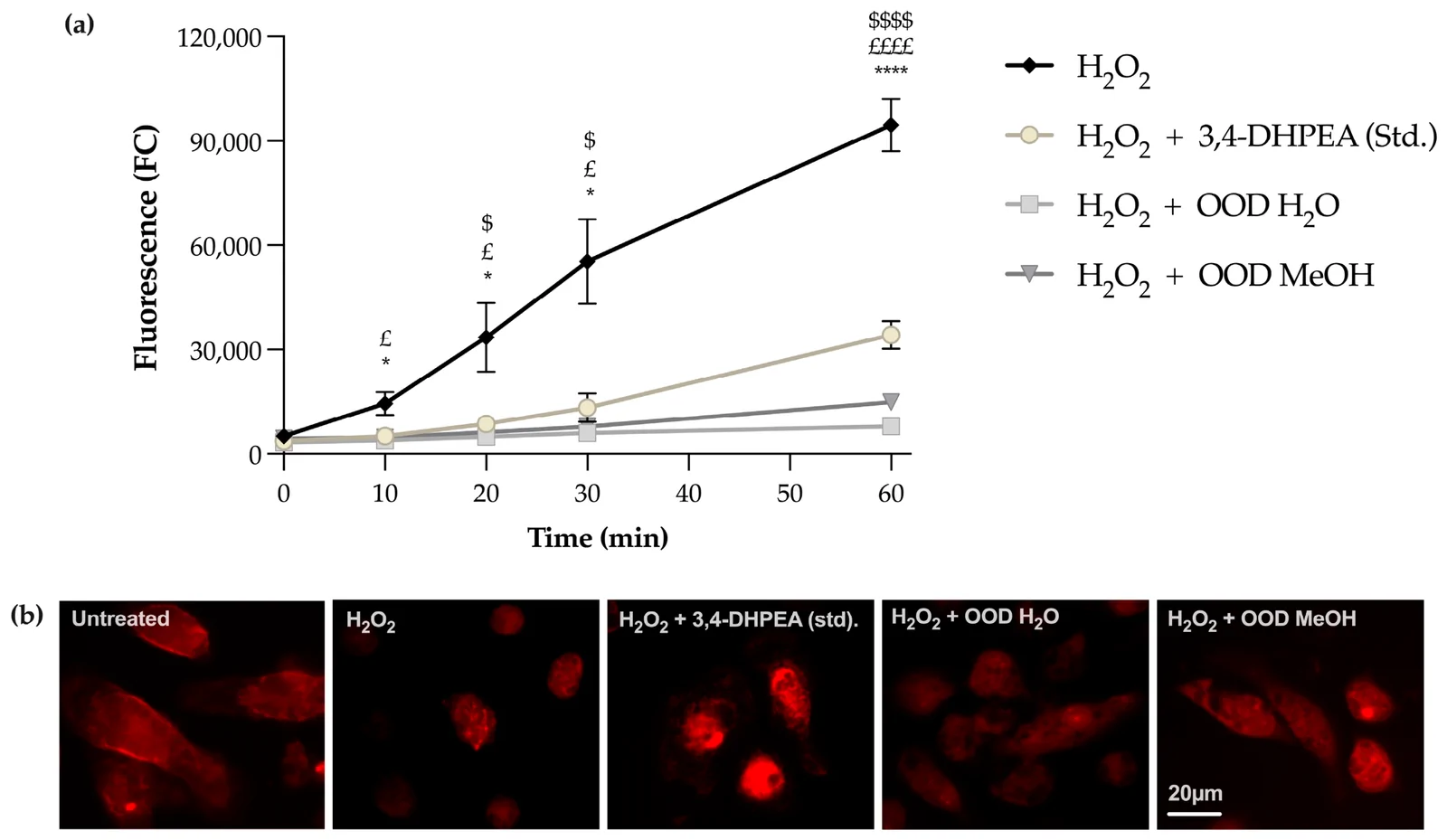
Intracellular ROS level reduction induced by OOD extracts. (**a**) Time course of intracellular ROS levels induced by H_2_O_2_. Data represent the mean ± SEM from three independent experiments performed in duplicate. Statistical significance was determined by comparing the H_2_O_2_ data (black line) with other conditions at the same timepoint. Statistical analysis was conducted using a one-way ANOVA followed by Tukey’s test (* *p* < 0.05, **** *p* < 0.0001 compared to OOD H_2_O, lighter grey line; £ *p* < 0.05, ££££ *p* < 0.0001 compared to OOD MeOH, darker grey line; $ *p* < 0.05, $$$$ *p* < 0.0001 compared to 3,4-DHPEA (Std.), yellow line. (**b**) Morphological analysis of murine peritoneal macrophages treated with H_2_O_2_ in the presence or absence of methanolic or aqueous OOD extracts, using phalloidin-Rhodamine staining. Fluorescence microscopy images were taken at 63× magnification. Scale bars, 20 µm.

To further evaluate the antioxidant activity of OOD extracts, intracellular ROS levels were measured in primary murine peritoneal macrophages. Both methanolic and aqueous OOD extracts, as well as the 3,4-DHPEA Std., were tested at a concentration of 10 μg/mL in the presence of ROS inducers. The experiments were conducted on adherent cells, where hydrogen peroxide (H_2_O_2_) at 450 μM was used as a positive inducer. As shown in Figure 2a, fluorescence signals, reflecting intracellular ROS levels, began to increase in the presence of H_2_O_2_ after 10 min. Fluorescence signal inhibition was observed, with statistical significance, in macrophages co-stimulated with both OOD extracts and 3,4-DHPEA Std., starting from 10 min.

At 1 h, the inhibitory effect was more pronounced with the OOD H_2_O extract compared to the OOD MeOH or 3,4-DHPEA standard at the corresponding time point. Collectively, these experiments demonstrate that both aqueous and methanolic OOD extracts effectively inhibit the accumulation of intracellular ROS. Notably, the aqueous extract exhibited a more pronounced antioxidant effect than the methanolic extract and the standard. Considering that 3,4-DHPEA is the predominant phenolic compound in our extracts and is recognized for its potent antioxidant properties, these results indicate that other phenolic constituents and/or synergistic interactions within the aqueous extract may enhance its activity beyond that of 3,4-DHPEA alone.

To further explore the protective potential of OOD extracts against oxidative damage, a morphological analysis of murine peritoneal macrophages was performed. Given that cytoskeletal alterations and membrane disruptions are indicative of oxidative stress, a fluorescent phalloidin staining assay was used to visualize cytoskeletal structural changes in response to an inflammatory stimulus. This approach therefore provided insights into the extracts’ modulatory effects on cell morphology under stress conditions. After 1 h of exposure to H_2_O_2_, phalloidin staining revealed that peritoneal macrophages acquired a contracted and rounded morphology, characterized by the presence of large cytoplasmic vacuoles, consistent with enhanced macropinocytosis occurring during the early contractile phase [19]. As shown in Figure 2b, cells treated with H_2_O_2_ exhibited a more vesicular morphology compared to untreated control cells. In contrast, co-treatment with both OOD extracts and standard attenuated the contractile rounding induced by H_2_O_2_, leading to a more elongated morphology closer to control cells. This analysis suggests that both extracts, irrespective of the extraction method, can mitigate the morphological alterations induced by oxidative stress, better than the standard. However, the formation of intracellular vacuoles, consistent with enhanced macropinocytosis, was not efficiently reduced, suggesting that antioxidant activity is more effective in restoring cytoskeletal morphology than in suppressing vesicular internalization processes already triggered by oxidative stress.

### 2.3. Aqueous Extract Inhibits RANKL-Induced Osteoclastogenesis

Early research in the 1990s identified oxidative stress as a key regulator of osteoclastogenesis, a hypothesis that has since been supported by extensive evidence [20]. ROS, such as superoxide and hydrogen peroxide, not only drive osteoclast precursor differentiation by enhancing intracellular signaling downstream of RANKL–RANK interaction but are also produced via RANKL-induced activation of NADPH oxidases (NOX), thereby reinforcing osteoclastogenic signaling in a feed-forward manner. Beyond their role in precursor differentiation, ROS also sustain the functional activity of mature osteoclasts, making redox modulation a promising strategy in limiting bone resorption [21]. Thus, targeting ROS has emerged as a promising strategy for preventing inflammatory and age-related bone diseases. Within this framework, we hypothesized that the antioxidant properties of OOD extracts could mitigate ROS-driven signaling, thereby inhibiting osteoclast formation and activity.

To this end, we employed the murine RAW 264.7 macrophage cell line, a well-established in vitro model for studying osteoclast differentiation [22]. Cells were treated with RANKL in the presence or absence of OOD extracts and were subsequently subjected to tartrate-resistant acid phosphatase (TRAP) staining to identify and count mature osteoclasts, defined as purple–pink TRAP-positive multinucleated cells containing three or more nuclei.

**Figure 3:**
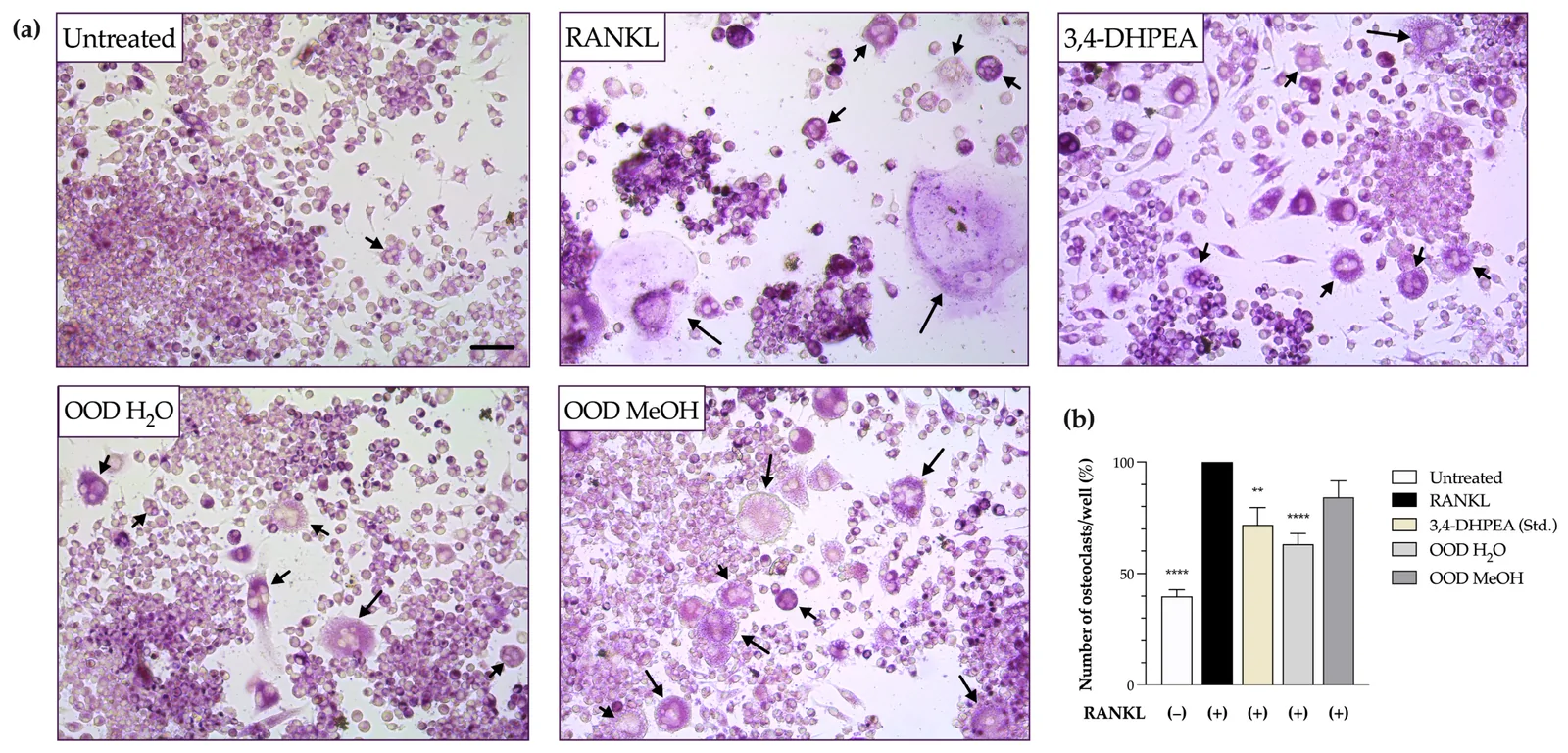
TRAP assay reveals inhibitory effects of OOD H_2_O on RANKL-induced osteoclast formation. RAW 264.7 cells were treated for 5 days with RANKL in the absence (black bar) or presence of test extracts or a reference standard (1 µg/mL; yellow bar). An untreated control was also included (white bar); (**a**) Representative images of TRAP (tartrate-resistant acid phosphatase) staining at 20× magnification; arrows indicate TRAP-positive multinucleated osteoclasts (OCs); a long arrow highlights a typical multinucleated giant cell. (**b**) Quantification of TRAP-positive OCs, defined as cells containing ≥3 nuclei. Data represent the mean ± SEM from three independent experiments performed in duplicate. Statistical analysis was performed using one-way ANOVA followed by Tukey’s post hoc test (** *p* < 0.01, **** *p* < 0.0001 vs. RANKL). Scale bar (**a**), 50 µm.

As shown in Figure 3a, in the untreated RAW 264.7 control group, cells maintained their typical macrophage-like morphology, appearing predominantly small, round, and mononuclear. By contrast, in the positive control stimulated with RANKL, cells exhibited evident morphological changes characteristic of osteoclast differentiation, including increased cell size, multinucleation, and irregularly shaped structures. Treatment with the extracts and 3,4-DHPEA standard completely abolished the formation of giant multinucleated cells and markedly reduced the population of big osteoclasts (>3 nuclei). The results, reported in Figure 3b, revealed that the aqueous OOD extract, at a concentration of 1 µg/mL, significantly inhibited RANKL-induced osteoclast formation. Similarly, treatment with 3,4-DHPEA std. resulted in a significant reduction in the number of OCs, an effect that may be attributed to its well-documented antioxidant properties through the preservation of mitochondrial function, as previously demonstrated by Zhang et al. [23]. Conversely, the methanolic extract did not show a comparable inhibitory effect under the same experimental conditions. Indeed, OOD MeOH treatment resulted in a high number of big osteoclasts, morphologically similar to those observed in the positive control, indicating continued osteoclast differentiation and, consequently, a limited inhibitory effect.

This suggests that the bioactive components responsible for osteoclastogenesis inhibition may be better preserved or more efficiently extracted in the aqueous preparation, or, alternatively, that the two extraction methods yield qualitatively distinct phytochemical profiles.

### 2.4. Aqueous Extract Impairs F-Actin Ring Formation in RANKL-Induced Osteoclasts

A tight F-actin ring is a prerequisite for osteoclastic bone resorption and is an observable marker of mature osteoclasts [24,25]. To further confirm the inhibitory effect on osteoclast differentiation, we analyzed and counted multinucleated cells with F-actin rings by immunofluorescence staining.

**Figure 4:**
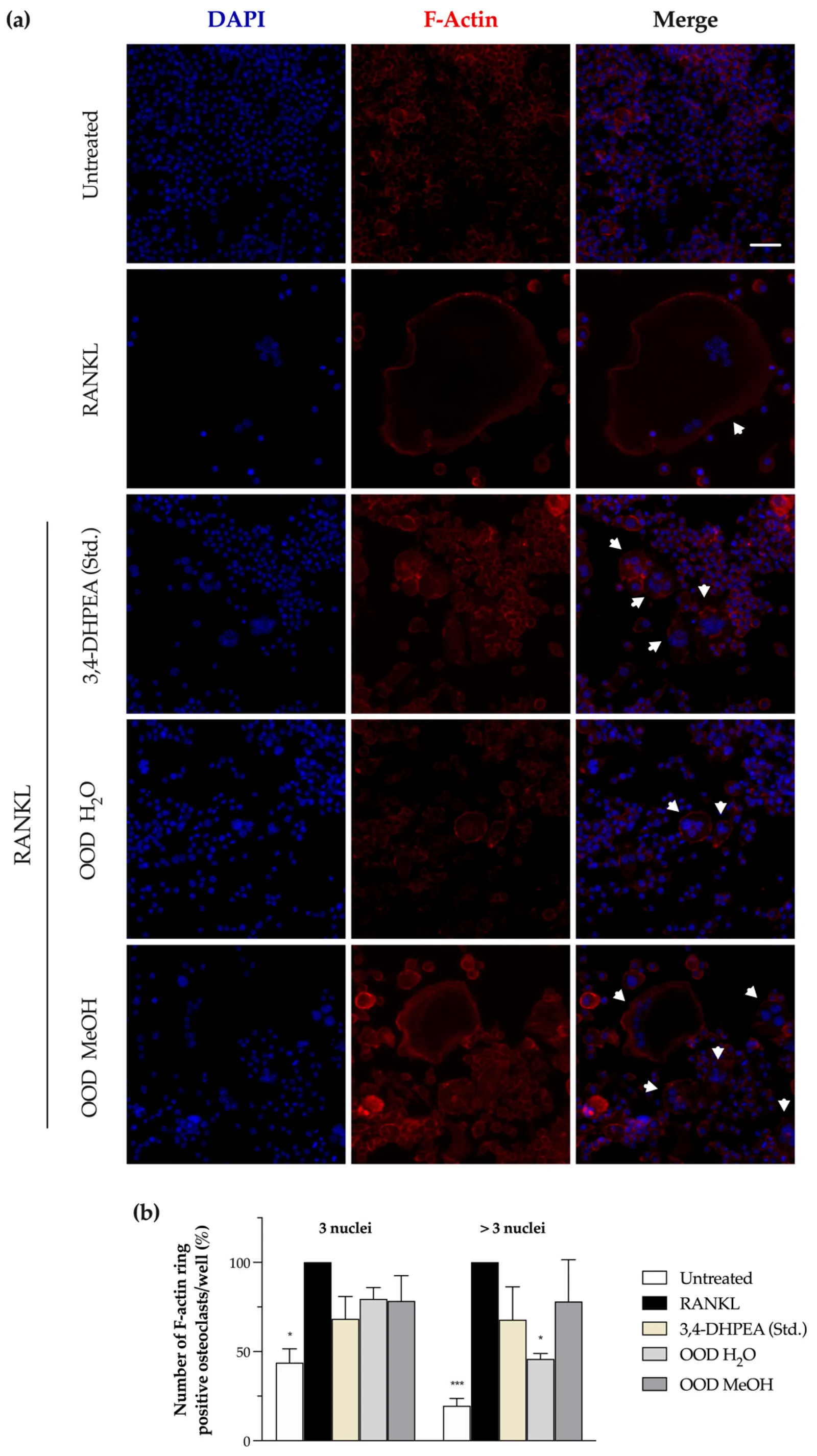
OOD H_2_O impairs F-actin ring formation in RANKL-induced osteoclasts. (**a**) Representative confocal microscopy images of RANKL-induced osteoclasts showing F-actin ring formation (red) visualized by phalloidin staining at 10× magnification. Nuclei were counterstained with DAPI (blue). Osteoclasts showing typical F-actin rings are indicated by white arrows. Scale bar, 50 µm. (**b**) Quantification of F-actin ring–positive multinucleated cells (≥3 nuclei). Data are expressed as the mean ± SEM from three independent experiments performed in duplicate. Statistical analysis was performed using two-way ANOVA followed by Tukey’s post hoc test (* *p* < 0.05, *** *p* < 0.001 vs. RANKL).

Figure 4a shows a representative panel of confocal microscopy images illustrating the organization of F-actin rings in RAW 264.7-derived osteoclasts following 5-day treatment with RANKL, in the presence or absence of test extracts. Mature osteoclasts were identified as large multinucleated cells (≥3 nuclei) exhibiting well-defined actin rings. In RANKL-treated cells, robust F-actin ring formation was observed, whereas co-treatment with OOD H_2_O resulted in a visible reduction in both the number and size of actin-positive multinucleated cells, indicating impaired osteoclast maturation.

Consistent with TRAP staining results, treatment with the aqueous extract at 1 µg/mL significantly reduced the number of large multinucleated osteoclasts (with >3 nuclei) with intact-structured F-actin rings, compared to cells treated with RANKL alone (Figure 4b). Smaller OCs (3 nuclei) appeared less affected. The OOD MeOH extract did not exhibit a comparable inhibitory effect on F-actin ring formation, indicating that this preparation was not as effective as the aqueous one in suppressing osteoclast maturation. The 3,4-DHPEA std. showed a trend toward reducing the number of F-actin–positive multinucleated OCs, although the effect did not reach statistical significance under the tested conditions.

These findings indicate that the aqueous OOD extract at 1 µg/mL effectively counteracts RANKL-dependent osteoclast differentiation, an effect likely linked, at least in part, to its antioxidant capacity and the synergistic action of multiple bioactive compounds beyond hydroxytyrosol alone.

Overall, the results obtained through morphological analysis using phalloidin and DAPI are consistent with the TRAP assay data, confirming that the aqueous extract from the olive oil production chain significantly interferes with osteoclast maturation, whereas the methanolic extract and 3,4-DHPEA standard show a more limited impact on the process of osteoclastogenesis.

### 2.5. Bone Resorption Pits

**Figure 5:**
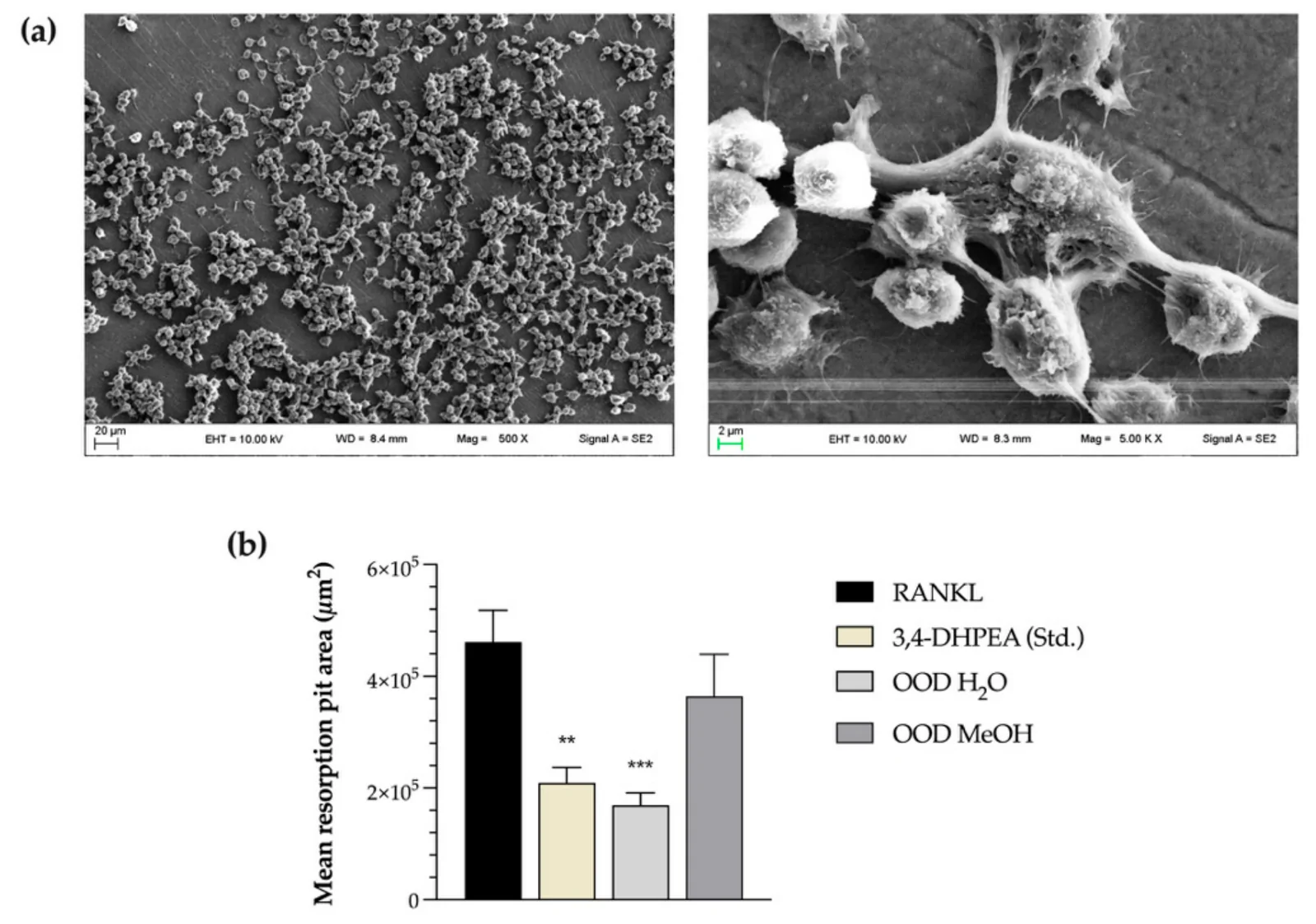
(**a**) SEM images. (**b**) Morphometric quantification of the pits. Statistical analysis was performed using one-way ANOVA followed by Tukey’s post hoc test (** *p* < 0.01, *** *p* < 0.001 vs. RANKL).

To evaluate the potential inhibitory effect of OOD extracts on osteoclast-mediated bone resorption, RAW 264.7 murine macrophages were seeded onto calcium phosphate (CaP)-coated substrates and induced to differentiate into osteoclasts in the presence of RANKL. After 5 days of culture, resorption pits generated on the mineralized surface were visualized by calcein staining and analyzed by fluorescence microscopy following Scanning Electron Microscopy acquisition. In Figure 5a are reported SEM representative images of undifferentiated RAW 264.7 (on the left) and the differentiated ones (on the right). When quantified by morphometric analysis of the pits, OCs activity showed extensive resorption of the bone surface by osteoclasts in the control group (RANKL treated RAW 264.7). Bone resorption area was significantly reduced to 42% and 77% after treatment with OOD-H_2_O and OOD-MeOH respectively; moreover, rare resorption pit was observed in the plate containing cells treated with 3,4-DHPEA (Std) (Figure 5).

### 2.6. Phenolic Compounds in Aqueous OOD Extract

**Figure 6:**
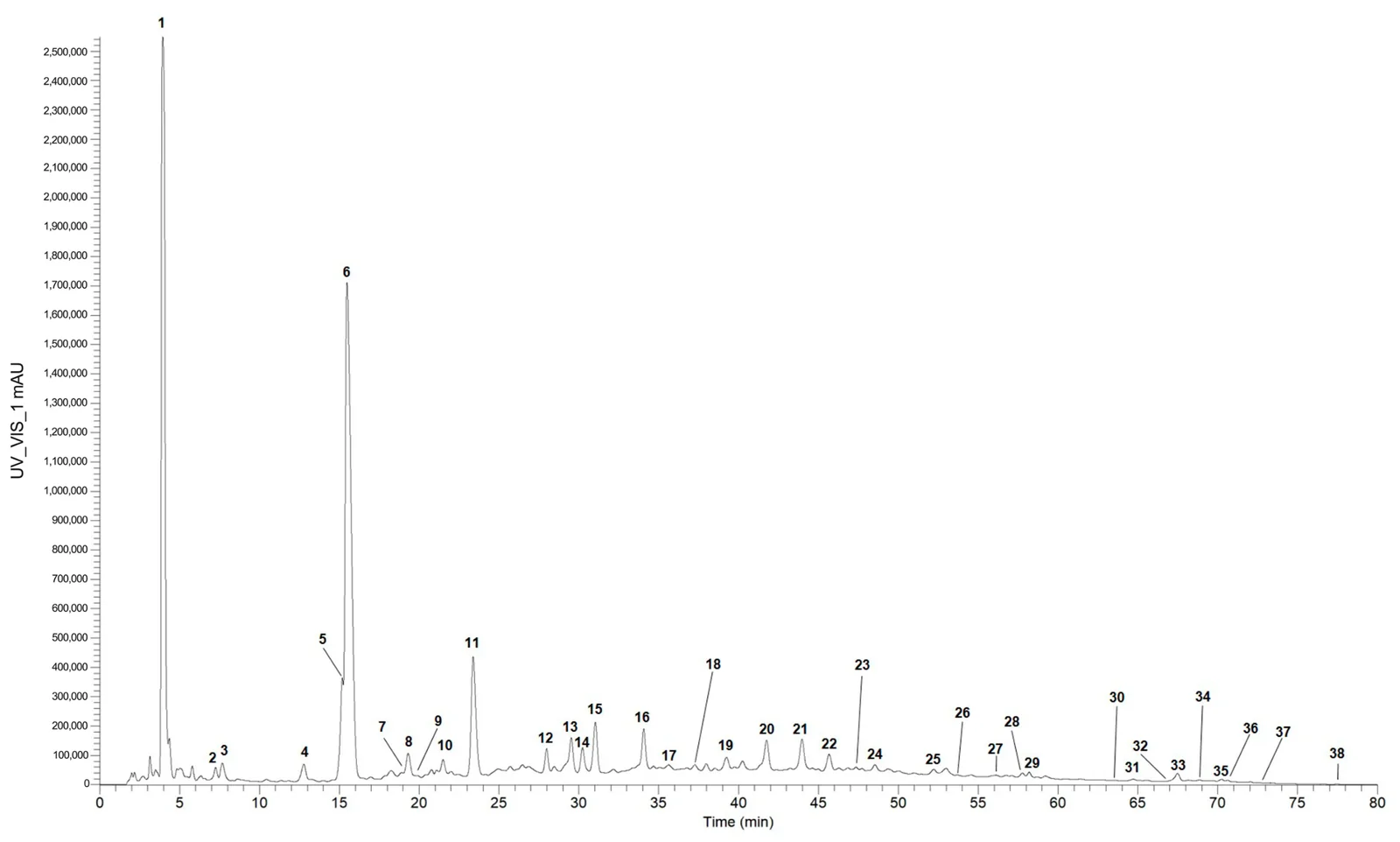
HPLC-UV chromatogram recorded at 280 nm showing phenolic compounds present in the aqueous OOD extract. For peak assignments, see Table 2.

Based on the results from the biological assays, analyses of phenolic compounds were subsequently conducted only on aqueous OOD extract using HPLC-UV/Vis and HPLC-ESI-ITMS^n^. The overall analysis led to the detection and tentative identification of 45 compounds based on the interpretation of their fragmentation patterns obtained from mass spectra (MS^n^ experiments) and by comparison with the literature. The classes of phenolic compounds detected in these analyses agreed with those already reported in previous studies on different parts of the olive tree (leaves, stems and fruit epicarp, mesocarp and seed), olive oils, and olive mill by-products [26,27,28,29,30,31,32], and included phenolic acids, phenolic alcohols, flavonoids, secoiridoids, and a lignan. Moreover, two lipids were also tentatively identified. The HPLC-UV-Vis chromatogram of compounds detected in aqueous OOD extract is shown in Figure 6.

HPLC/ESI-ITMS^n^ analyses of the aqueous OOD extract showed a compound with pseudomolecular ion [M–H]^−^ at *m*/*z* 243 in peak 21 (Figure 6), displaying a Δ *m*/*z* = +2 compared to the [M–H]^−^ of elenolic acid, coherent with a reduced (hydrogenated) form of elenolic acid. Moreover, the MS2 spectrum revealed fragment ions at *m*/*z* 211 and 165 due to the loss of CH_3_OH [M–H-32]^−^, followed by a subsequent loss of CO_2_ [M–H-32-44]^−^. This fragmentation pattern was consistent with that reported for elenolic acid. The presence of hydrogenated elenolic acid has previously been reported in fermented olive leaves and olive oil [33].

Peaks 28, 30 and 31 showed the same pseudo-molecular ion (*m*/*z* 555) (Figure 6) and were tentatively identified as 6′-O-[(2E)2,6-Dimethyl-8-hydroxy-2-octenoyloxy]-secologanoside derivatives based on the mass spectral data reported in a previous study on olive pomace [34]. Moreover, in the mass spectrum of peak 31, an ion at *m*/*z* 319 was also present and was identified as decarboxymethyl oleuropein aglycone. The list of all compounds identified in aqueous OOD extract is reported in Table 2.

## 3. Materials and Methods

### 3.1. Sample Preparation and Chemical Analyses

#### 3.1.1. Olive Oil Dregs (OOD) and Extraction Procedures

Olive oil dregs (OOD) were generously supplied by EVO Campania, an olive oil mill situated in southern Italy (Campagna, Salerno). Upon receipt, the sample was aliquoted, stored at −80 °C to prevent degradation, and thawed prior to use. In the present study, two different methods for the preparation of polyphenol-rich extracts from OOD were applied in accordance with Squillaci et al., 2021 [16]. In the first method, OOD was mixed with n-hexane (1:4 *w*/*v*). After 20 min of stirring, the n-hexane was removed, and the residue was mixed with methanol/1% HCl (70/30) in a 1:1 ratio and incubated at 37 °C for 30 min under stirring. In the second method, OOD was mixed with acidified water (pH 1.25) (1:2 *w*/*v*) and stirred at 37 °C for 60 min. Both methanol and aqueous phases were recovered by centrifuging the extraction mixtures at 13,200 rpm at 4 °C for 60 min. The supernatants were further clarified by filtration with a 0.45 μm pore size filter and for chemical analysis the methanol and aqueous extracts were stored at 4 °C until analyzed. Following the initial extraction process, for the biological tests both the aqueous and methanolic extracts were thoroughly dried (evaporated to dryness) to completely remove the original solvents. The resulting dry residues were then reconstituted and diluted in the exact same vehicle. This step ensures a fair and direct comparison between the biological properties of the two extracts, entirely independent of the initial solvent environment. In parallel, to definitively rule out any residual solvent influence or toxic side-effects on the cells, we included appropriate vehicle blank probes (containing the respective vehicle controls without any active substances) in all biological assays.

#### 3.1.2. Quantification of Hydroxytyrosol by Reverse-Phase High-Performance Liquid Chromatography (RP-HPLC)

The hydroxytyrosol (3,4-DHPEA) content in both methanolic and aqueous OOD extracts was measured by reversed-phase high-performance liquid chromatography (RP-HPLC). A Dionex Ultimate 3000^®^ HPLC system (Thermo Fisher Scientific, Waltham, MA, USA) equipped with a quaternary pump and an Ultimate 3000^®^ Diode Array Detector (Thermo Fisher Scientific, Waltham, MA, USA) was used. Samples were filtered through a 0.22 µm pore-size membrane before injection into a Phenomenex Luna C18 (2) column (250 × 4.6 mm, 5.0 µm), protected by a SecurityGuard™ pre-column containing a C18 cartridge (Phenomenex, Torrance, CA, USA). 3,4-DHPEA separation was performed under the following conditions: a constant flow rate of 700 µL/min; solvent A consisted of 0.5% acetic acid in degassed ultrapure water, and solvent B was a degassed mixture of ultrapure water and acetonitrile (1:1, *v*/*v*) containing 0.1% acetic acid. The elution program was set as follows: 0–5 min, 5% B (isocratic); 5–60 min, linear gradient to 55% B; 60–70 min, linear gradient to 95% B; followed by a 10 min hold at 95% B. 3,4-DHPEA was detected at 280 nm. 3,4-DHPEA identification was achieved by comparing the retention time and absorption spectrum with those of a pure commercial standard (Sigma-Aldrich). Quantification of 3,4-DHPEA, expressed as µg of 3,4-DHPEA, was performed using a calibration curve (y = 26.862x; r^2^ = 0.9975) generated by injecting increasing amounts of 3,4-DHPEA standard solution. The standard compound was prepared at a concentration of 1 mg/mL in ultrapure water, and the calibration curve was constructed by analyzing four standard injections corresponding to 0.3, 1.0, 2.5, and 5.0 µg of 3,4-DHPEA.

#### 3.1.3. Analysis of Phenolic Compounds Contained in Aqueous OOD Extract by Ultra-High-Performance Liquid Chromatography–Multi-Stage Mass Spectrometry (UHPLC-MS^n^) and Reversed-Phase High-Performance Liquid Chromatography–Ultraviolet (RP-HPLC–UV) Analyses

Samples of aqueous OOD extract were analyzed by ultra-high performance liquid chromatography (UHPLC)-linear ion trap mass spectrometry on an LTQ XL ion-trap mass spectrometer coupled with a UHPLC Ultimate 3000 RS chromatographic system equipped with a Diode Array Detector and Xcalibur^®^ system manager data acquisition software v4.5 (Thermo Fisher Scientific, Waltham, MA, USA). Individual compounds were separated on a Phenomenex Luna C18 (2) column (100 Å, 150 × 2.0 mm, 5.0 µm, Phenomenex, Torrance, CA, USA) at a flow rate of 200 μL min^−1^. The HPLC separation solvents and the elution program are described in the previous paragraph. The column effluent was split into two by means of a “T junction” placed after the chromatographic column and analyzed “on-line” both by UV-Vis and heated electrospray ionization mass spectrometry (HESI/MS); 80% of the effluent was sent to the Diode Array Detector (wavelength range 200–800 nm) while 20% of the effluent was analyzed by HESI/MS. Mass spectra were recorded from *m*/*z* 120 to 2000 in negative ionization mode. The capillary voltage was set at −27 V, the spray voltage was at 4 kV and the tube lens offset was at −87 V. The heater temperature was set at 200 °C and the capillary temperature was 275 °C. Data were acquired in MS and MS^n^ scanning mode. All solvents were of HPLC grade, HPLC grade water (18.2 mΩ) was prepared using a Millipore Milli-Q purification system (Millipore Corp., Bedford, MA, USA).

#### 3.1.4. Total Antioxidant Capacity

The total antioxidant capacity of the extracts was determined using the MAK187-1KT kit (Sigma-Aldrich, St. Louis, MO, USA), based on the reduction of Cu^2+^ to Cu^+^, which forms a colorimetric complex detectable at 570 nm. Samples and Trolox standards (0–20 nmol/well) were mixed 1:1 with the Cu^2+^ working solution in a microplate and incubated for 90 min at room temperature while being protected from light. Absorbance was measured at ~570 nm using a Viktor X4 spectrophotometer (PerkinElmer, Shelton, CT, USA). The concentrations of 1 μg/mL and 10 μg/mL for the TEAC assay were selected based on previous cytotoxicity screenings on osteoblasts, where higher concentrations exhibited toxic effects [35,36]. Therefore, the antioxidant activity was evaluated exclusively within this biologically safe and relevant range to justify their use in subsequent cell culture experiments.

### 3.2. Biological Assays

#### 3.2.1. Cell Cultures

Peritoneal macrophages, used in 4.2.2, were isolated from wild-type C57BL/6 mice (Charles River Laboratories, Wilmington, MA, USA), provided by the Department of Translational Medicine, University of Eastern Piedmont. Animal care and husbandry were in conformity with the institutional guidelines in compliance with national (D.L. n. 26/2014) and international laws and policies (EEC Council Directive 86/609, OJ L358, 1, 12 December 1987; Guide for the Care and Use of Laboratory Animals, U.S. National Research Council, 2011). The mice used for the macrophages test were BALB/c mice aged 4–10 weeks from Charles River Laboratories International, Inc. The study plan and the procedures were approved by the University of Piemonte Orientale and authorized by the Italian Ministry of Health (authorization number DB064.N.QCY and 449/2020-PR, approval date 24 October 2023). In total, six animals were used (two for each of the three independent experiments). The mice were anesthetized under deep isoflurane-induced anesthesia. All efforts were made to reduce the number of animals by following the 3R’s rule. Animals were housed under a controlled environment with constant humidity, temperature (20–22 °C) and a 12 h light cycle, as well as with unlimited access to food and water, and lights turned off from 7.00 p.m. to 7.00 a.m. Peritoneal cavities were flushed with 5 mL of cold PBS and gently massaged (10–15 s) to collect cells. The cell suspension was kept at 4 °C, centrifuged at 1000 g for 5 min, washed, and resuspended in RPMI medium supplemented with 20% fetal bovine serum (FBS, Euroclone, Pero, Italy). Cells were cultured at 37 °C in a humidified 5% CO_2_ incubator.

The murine macrophage cell line RAW 264.7 was obtained from the American Type Culture Collection (ATCC, Manassas, VA, USA). Cells were cultured in RPMI-1640 medium supplemented with 10% heat-inactivated fetal bovine serum (FBS, Gibco, Thermo Fisher Scientific, Grand Island, NY, USA), 2 mM L-glutamine (L-gln), and antibiotics (100 U/mL penicillin, 100 μg/mL streptomycin; P/S).

#### 3.2.2. Intracellular ROS Detection and Morphological Analysis

Intracellular reactive oxygen species (ROS) were quantified using the DCFDA/H2DCFDA kit (ab113851, Abcam Ltd., Cambridge, UK). Adhered peritoneal macrophages (2.5 × 10^4^ cells/well) were incubated with 20 µM DCFDA dye solution for 45 min at 37 °C and 5% CO_2_ in the dark. After incubation and dye removal, cells were treated with 1× Buffer—10% FBS, with or without extracts or an oxidative stress inducer (H_2_O_2_ 50 mcM). Fluorescence was measured at 485/535 nm using a Viktor X4 plate reader (PerkinElmer, Shelton, CT, USA). For morphological analysis, adherent macrophages were fixed with 4% formalin for 30 min at room temperature and then stained with rhodamine-conjugated phalloidin (10 µg/mL, Sigma-Aldrich) for 60 min at 37 °C in the dark to visualize actin filaments. Fluorescent images were acquired using a Leica DMRB fluorescence microscope (Wetzlar, Germany) equipped with a digital camera.

#### 3.2.3. TRAP Staining

RAW 264.7 cells were seeded at a density of 3 × 10^3^ cells/well in a flat-bottom 96-well plate and incubated overnight for cell attachment. The day after seeding, cells were induced to differentiate into osteoclasts (OCs) by treatment with RANKL (100 ng/mL) in the presence or absence of test extracts. Differentiation was carried out for 5 days, with medium refreshed on day 3. At the end of the treatment period, cells were fixed and stained using a solution composed of 2.5 M acetate buffer (pH 5.2), Red Fast Violet (0.8 mg/mL) and NABP AS-BI (0.1 mg/mL). Stained cells were imaged using a bright-field light microscope (Leica DMi1, Leica Microsystems GmbH, Wetzlar, Germany) at 20× magnification. TRAP-positive multinucleated cells containing three or more nuclei were identified as osteoclasts and counted per well. All reagents were purchased from Sigma-Aldrich (St. Louis, MO, USA).

#### 3.2.4. F-Actin Ring Formation Assay

RAW 264.7 cells were seeded (3 × 10^3^ cells/well) in 96-well plates and treated according to the previously outlined protocol. After 5 days, cells were fixed with 4% paraformaldehyde (PFA) for 30 min at room temperature. F-actin ring formation was assessed by staining cells with rhodamine-conjugated phalloidin (10 µg/mL; Sigma-Aldrich) for 60 min at 37 °C in the dark. Nuclei were subsequently stained with 4′,6-diamidino-2-phenylindole (DAPI) (40 µM; Sigma-Aldrich). Fluorescent images were acquired using an LSM700 laser-scanning confocal microscope (Carl Zeiss Microscopy GmbH, Jena, Germany). F-actin ring–positive cells containing three or more nuclei were identified as osteoclasts and counted per well.

#### 3.2.5. Bone Resorption Assay

RAW 264.7 cells were seeded at a density of 1 × 10^4^ cells/well in 24-well plates pre-coated with calcium phosphate (CaP) (COSMO BIO Co., Ltd., Tokyo, Japan), following the manufacturer’s instructions. After overnight adhesion, cells were induced to differentiate into osteoclasts in the presence of RANKL and the tested extracts. After 10 days of culture, with re-stimulation every 3 days, the cells were fixed with 4% paraformaldehyde (PFA) for 30 min at room temperature. The mineralized CaP substrate was incubated with calcein solution (5 µg/mL; Sigma-Aldrich) to detect resorption pits. Fluorescence images of resorption lacunae were acquired using a Leica DMRB fluorescence microscope (Wetzlar, Germany) with 5× magnification, and quantitative analyses of pit area were performed with ImageJ software, version 1.53t (NIH, Bethesda, MD, USA). Experiments were performed in triplicate, with seven areas (2 mm^2^ each) measured per experiment (*n* = 21).

#### 3.2.6. Scanning Electron Microscopy

For ultrastructural analysis, RAW 264.7 cells or RAW cells differentiated with RANKL to osteoclasts were cultured in contact with the bone substitute materials for 5 days. At the end of the culture period, samples were gently rinsed in a cacodylate buffer (0.1 M, pH 7.4) to remove unattached cells and debris and subsequently fixed with 1.5% glutaraldehyde in the same buffer for 15 min at room temperature. Following fixation, samples were washed three times in cacodylate buffer and dehydrated through a graded ethanol series (70%, 80%, 95%, and 100%; 5 min each step). To preserve cellular morphology, samples were then dried using hexamethyldisilazane. The dried specimens were mounted onto aluminum stubs, sputter-coated with a thin layer of chromium (~10 nm) and examined using an environmental scanning electron microscope (Zeiss EVO-MA10 scanning electron microscope, Carl Zeiss, Oberkochen, Germany) operated at an accelerating voltage of 10 kV.

#### 3.2.7. Statistical Analysis

Quantitative data are expressed as the mean ± standard error of the mean (SEM) from a minimum of three independent experiments. Statistical analysis was performed using one-way ANOVA followed by Tukey’s post hoc test. A *p*-value of <0.05 was considered statistically significant. GraphPad Prism software (version 10) was used for all analyses.

## 4. Discussion and Conclusions

An imbalance in bone homeostasis caused by increased osteoclast number and/or activity can lead to pathological bone resorption, a hallmark of several osteolytic diseases, including rheumatoid arthritis, periprosthetic osteolysis, periodontitis and osteoporosis [37]. Consequently, osteoclasts represent an important therapeutic target for limiting excessive bone loss. Over the past two decades, substantial progress has been made through osteoclast-targeting therapies, including estrogens, bisphosphonates, cathepsin K inhibitors and denosumab. However, their long-term use can be associated with adverse effects, limiting their clinical application and highlighting the need for alternative strategies with improved safety profiles [38].

To our knowledge, the present study is among the first to investigate the anti-osteoclastogenic potential of OOD. OOD markedly reduced RANKL-induced osteoclast formation and bone resorption at low concentrations without compromising cell viability, indicating that the observed effect was not attributable to cytotoxicity. To ensure that the observed biological effects were not biased by cellular stress, cell viability was systematically monitored in parallel via MTT assay. In agreement with the previous literature establishing the toxicity threshold of hydroxytyrosol well above 10 µM [39,40,41], our results confirmed the complete absence of cytotoxicity for both short-term (10 µM) and long-term (1 µM) treatments (data available upon request). Importantly, this response was accompanied by a strong antioxidant capacity by mitigating H_2_O_2_-induced ROS accumulation in primary peritoneal macrophages. Although we did not directly quantify the intracellular ROS kinetics triggered specifically by RANKL stimulation—which represents a limitation of the current setting due to primary cell availability and ethical constraints—our findings strongly suggest an interplay between the compound’s scavenger activity and impaired osteoclastogenesis. Indeed, RANKL signaling triggers an endogenous wave of ROS that acts as a mandatory second messenger for cytoskeletal remodeling and maturation. The fact that OOD comprehensively suppressed not only TRAP activity and giant cell fusion, but also actin ring formation and functional bone resorption (pit formation), strongly implies that its antioxidant action successfully disrupts the upstream signaling cascade required for full osteoclast activation. This aligns with the previous literature showing that targeted ROS scavenging blunts the entire functional program of mature osteoclasts. ROS are increasingly recognized as important signaling mediators during RANKL-induced osteoclastogenesis. For example, work by Kim et al. [42] showed that sustained RANKL-induced ROS production promotes Ca^2+^ oscillations and NFATc1 activation, hence supporting the late stages of osteoclastogenesis and bone resorption. Therefore, ROS production can favor osteoclast formation and bone-resorptive activity, whereas antioxidant compounds may attenuate this process by limiting oxidative signaling. Lee et al. [43] demonstrated that RANKL stimulates intracellular ROS production through a TRAF6–Rac1–Nox1-dependent pathway, and that inhibition of ROS generation using Trolox, a vitamin E analog with antioxidant activity, suppresses MAPK activation and osteoclast differentiation, thereby potentially limiting excessive bone resorption. The reduction in ROS observed following OOD treatment is therefore consistent with its inhibition of osteoclast formation and resorptive activity.

Collectively, these findings identify OOD as a potentially valuable source of bioactive compounds capable of modulating RANKL-induced osteoclastogenesis and oxidative stress. Beyond providing a potential strategy for controlling excessive bone resorption, the valorization of this agri-food by-product may offer an additional route for transforming olive oil processing residues into biologically functional materials.

The antiosteoclastogenic effect of OOD may be tentatively attributed to multiple active compounds, considering that the antioxidant activity was not directly proportional to the 3,4-DHPEA percentage in the sample. These results suggest the possibility that other substances within the complex mixture may contribute to or modulate this activity, a hypothesis that warrants further investigation to formally test for synergistic effects.

HPLC analysis of the crude extract, identifying in addition to 3,4-DHPEA other molecules with known antioxidant and anti-osteoclastogenesis activity, confirms our hypothesis. Among these, phenolic and organic acids (gallic, vanillic, quinic, and elenoic acids) primarily downregulate osteoclastogenesis by suppressing RANKL-induced signaling pathways (such as NF-κB, MAPK/ERK, and Akt) and reducing the expression of key osteoclastic markers like NFATc1 and TRAP [44]. Concurrently, the 18-carbon unsaturated fatty acids (octadecenoic (oleic) and octadecadienoic (linoleic) acids) act as crucial negative regulators. They modulate lipid-sensing receptors (e.g., GPR120) and attenuate inflammatory cascades, effectively impairing mature osteoclast formation and function. Together, this specific combination of phytochemicals and fatty acids offers a potent synergistic potential to shift bone remodeling dynamics toward an osteoprotective state [10]. Moreover, oleuropein aglycone and oleocanthal exert strong osteoprotective effects; oleuropein aglycone shifts bone marrow stem cell differentiation away from osteoclastogenesis by optimizing the OPG/RANKL ratio, while oleocanthal suppresses inflammatory cytokines (such as IL-6 and TNF-α) via the NF-κB pathway to halt osteoclast maturation. Similarly, the lignan pinoresinol directly impairs RANKL-induced osteoclastogenesis by disrupting f-actin ring formation and downregulating key osteoclastic markers like TRAP and NFATc1 [45]. Furthermore, the iridoid glycosides secologanoside and osteoside actively mitigate bone loss by acting as anti-inflammatory and antioxidant agents, effectively blocking the transcriptional signals required for monocyte-to-osteoclast fusion [46]. Together, these molecules represent a diverse phytochemical matrix capable of attenuating pathologic bone resorption.

Overall, the data demonstrates that the effect of food-derived products on bone metabolism depends critically on the interplay between chemical composition, extraction method, and the activation state of osteoclastogenic signaling pathways. This study highlights the importance of integrating chemical characterization with functional assessment to gain a rational understanding of the biological potential of food-like extracts. Further studies investigating the underlying molecular pathways and validating these effects in more physiologically relevant models will be required to establish their therapeutic potential, as current evidence is limited to in vitro studies and awaits validation by in vivo experiments.

## Figures and Tables

**Table 1:** 3,4-DHPEA content in OOD extracts.

Extraction Method	3,4-DHPEA (µg/g OOD)
Acidified methanol (37 °C—30 min)	519.86 ± 9.08
Acidified water (37 °C—60 min)	534.40 ± 9.05

3,4-DHPEA: Hydroxytyrosol; OOD: Olive Oil Dregs.

**Table 2:** List of phenolic compounds identified in an aqueous OOD extract by HPLC-UV/Vis and HPLC-ITMS^n^: Specific quasi-molecular ions and fragment ions are reported for each compound.

Peak	tR (min)	[M–H]^−^ *m*/*z*	MS^n^ Ions *m*/*z*	Proposed Structure
**1**	3.95	191 169 151	MS^2^ [191]: 173, 127, 111 MS^2^ [169]: 151, 125 MS^2^ [151]: 123	Quinic acid Gallic acid Oxidized hydroxytyrosol (oxidized 3,4-DHPEA)
**2**	7.26	393	MS^2^ [393]: 375, 363, 331, 213, 151 MS^3^ [393→363]: 345, 319, 201, 183, 139 MS^3^ [393→375]: 357, 331, 195, 179, 161, 151	Unknown
**3**	7.68	183	MS^2^ [183]: 165, 139, 95	Dialdehydic form of decarboxymethyl elenolic acid (EDA)
**4**	12.78	201	MS^2^ [201]: 183, 157	Hydrated product of the dialdehydic form of decarboxymethyl elenolic acid (Hydrated product of EDA)
**5**	15.19	185	MS^2^ [185]: 167, 141, 111, 95	Hydrogenated decarboxymethyl elenolic acid Isomer 1
**6**	15.49	153	MS^2^ [153]: 123	Hydroxytyrosol (3,4-DHPEA)
**7**	18.90	241	MS^2^ [241]: 209, 165, 139, 127, 121, 101, 95	Elenolic acid (EA)
**8**	19.31	187	MS^2^ [187]: 169, 125, 95	Hydroxygallic acid
**9**	19.89	215	MS^2^ [215]: 197, 171	Aldehydic form of decarboxymethyl elenolic acid
**10**	21.50	199	MS^2^ [199]: 181, 155, 111 MS^3^ [199→155]: 137, 127, 111, 95 MS^3^ [199→181]: 153, 135, 121, 11, 93	Hydroxylated product of the dialdehydic form of decarboxymethyl-elenolic acid Isomer 1
**11**	23.38	185	MS^2^ [185]: 167, 141, 111, 95	Hydrogenated decarboxymethyl elenolic acid Isomer 2
**12**	27.98	199	MS^2^ [199]: 181, 155, 111 MS^3^ [199→155]: 137, 127, 111, 95 MS^3^ [199→181]: 153, 135, 121, 11, 93	Hydroxylated product of the dialdehydic form of decarboxymethyl-elenolic acid Isomer 2
**13**	29.52	167	MS^2^ [167]: 152, 123, 108	Vanillic acid
**14**	30.23	303 389	MS^2^ [303]: 285, 273, 259, 255, 243, 165, 163,151 MS^3^ [303→255]: 240, 237, 227, 211, 197, 145, 133, 109 MS^3^ [303→273]: 255, 243, 241, 225, 151 MS^2^ [389]: 345, 209, 183, 165, 139, 121	Tyrosol- dialdehydic form of decarboxymethyl elenolic acid isomer 1 (p-HPEA-EDA; Oleocanthal) Oleoside or secologanoside
**15**	31.03	365	MS^2^ [365]: 347 MS^3^ [365→347]: 315, 303, 287, 285, 273, 271, 255, 253, 243, 241, 227, 225, 211, 197, 183, 137	Dimethyl acetal of oleacein (dimethyl acetal of 3,4-DHPEA-EDA)
**16**	34.06	303	MS^2^ [303]: 285, 273, 259, 255, 243, 165, 163,151 MS^3^ [303→255]: 240, 237, 227, 211, 197, 145, 133, 109 MS^3^ [303→273]: 255, 243, 241, 225, 151	Tyrosol- dialdehydic form of decarboxymethyl elenolic acid isomer 2 (p-HPEA-EDA; Oleocanthal)
**17**	35.57	377	MS^2^ [377]: 197, 153	Oleuropein aglycone (3,4-DHPEA-EA)
**18**	37.26	257	MS^2^ [257]: 239, 225, 213, 211, 195, 181, 169, 153, 137, 111	Hydroxylated form of elenolic acid (Hydroxylated form of EA)
**19**	39.24	403	MS^2^ [403]: 371, 305, 241, 223, 179	Elenolic acid glucoside (EA glucoside)
**20**	41.75	375	MS^2^ [375]: 357, 333, 329, 311, 287, 255	Dehydro oleuropein aglycone Isomers 1
**21**	43.97	375 243 195	MS^2^ [375]: 357, 333, 329, 311, 287, 255 MS^2^ [243]: 211, 167, 123 MS^3^ [243→211]: 183, 151, 139, 123, 121, 111, 95 MS^2^ [195]: 151, 135, 107 MS^3^ [195→151]: 133, 123, 121, 107, 95, 79	Dehydro oleuropein aglycone Isomers 2 Hydrogenated elenolic acid (hydrogenated EA) Hydroxytyrosol acetate.
**22**	45.67	593	MS^2^ [593]: 473, 447, 285	Luteolin-C-glucoside-O-rhamnoside
**23**	47.35	593	MS^2^ [593]: 447, 285	Luteolin rutinoside
**24**	48.53	335	MS^2^ [335]: 317, 305, 291, 273, 247, 229	Methyl hemiacetal of oleocanthal (Methyl hemiacetal of p-HPEA-EDA
**25**	52.21	509	MS^2^ [509]: 491, 465, 179	Demethyl ligstroside
**26**	53.39	551	MS^2^ [551]: 533, 507, 389, 341, 281, 251, 221	Caffeoyl-6′-secologanoside (Cafselogoside)
**27**	56.11	335	MS^2^ [335]: 199, 181, 153, 121, 111 MS^3^ [335→199]: 181, 155, 153, 129, 121, 111, 95, 85.	10-Hydroxy-decarboxymethyl oleuropein aglycone
**28**	57.77	555	MS^2^ [555]: 511, 389, 345, 225 MS^3^ [555→511]: 493, 479, 451, 345, 327, 285, 255, 225, 197, 183, 167. MS^3^ [555→345]: 301, 207, 183, 179, 165, 143, 137, 125, 121, 119, 113, 101.	6′-O-[(2E) 2,6-Dimethyl-8- hydroxy- 2-octenoyloxy]-secologanoside derivative
**29**	58.19	535	MS^2^ [535]: 517, 491, 473, 389, 345, 325, 307, 265, 235, 209, 163	Comselogoside
**30**	63.54	555	MS^2^ [555]: 511, 389, 345, 225 MS^3^ [555→511]: 493, 479, 435, 345, 327, 285, 255, 225, 197, 183, 167. MS^3^ [555→345]: 301, 223, 183, 179, 165, 143, 137, 125, 121, 119, 113, 101.	6′-O-[(2E) 2,6-Dimethyl-8- hydroxy- 2-octenoyloxy]-secologanoside derivative
**31**	64.70	319 555	MS^2^ [319]: 181, 153, 111 MS^3^ [319→181]: 163, 153, 137, 135, 121, 111 MS^2^ [555]: 511, 389, 345. MS^3^ [555→511]: 493, 479, 435, 345, 327, 285, 255, 225, 197, 183, 167. MS^3^ [555→345]: 301, 223, 183, 179, 165, 143, 137, 125, 121, 119, 113, 101.	Decarboxymethyl oleuropein aglycone 6′-O-[(2E)-2,6-dimethyl-8-hydroxy-2-octenoyloxy]-secologanoside derivative
**32**	66.73	557	MS^2^ [557]: 513, 389, 345, 199, 185, 165 MS^3^ [557→513]: 481, 419, 345, 327, 257, 227, 209, 199, 185, 183, 179, 165, 161. MS^3^ [557→345]: 183, 179, 165, 161, 149, 143, 131, 121, 119, 113, 101.	6′-O-[2,6-dimethyl-8-hydroxy2-octenoyloxy]-secologanoside
**33**	67.49	357	MS^2^ [357]: 339, 315, 311 MS^3^ [357→311]: 293, 283, 279, 269, 267, 251, 237, 223, 209, 197, 183.	Pinoresinol
**34**	69.00	393	MS^2^ [393]: 361, 349, 317, 257, 241, 181, 137 MS^3^ [393→317]: 289, 247, 181, 153, 137, 109.	10-Hydroxy oleuropein aglycone
**35**	70.22	329 361	MS^2^ [329]: 311, 309, 293, 229 MS^3^ [329→311]: 293, 291, 249, 209, 195, 185, 171, 153, 139, 125, 113. MS^3^ [329→229]: 211, 209, 183, 167, 155, 127, 125, 97, 85. MS^2^ [361]: 343, 329, 325, 291, 273, 259, 229, 201, 193, 181, 149, 123. MS^3^ [361→343]: 325, 315, 307, 299, 285, 271, 237, 201, 197, 185, 171, 155, 137, 115. MS^3^ [361→181]: 165, 153, 149, 139, 123, 109, 95.	Trihydroxy-octadecenoic acid Ligstroside aglycone.
**36**	70.62	327 655	MS^2^ [327]: 309, 291, 239, 229, 221, 171 MS^3^ [327→309]: 291, 273, 263, 249, 247, 227, 209, 185, 171, 165, 155, 139, 125, 119, 95 MS^3^ [327→291]: 273, 247, 209, 193, 185, 179, 165, 163, 153, 149, 135, 123, 111 MS^3^ [327→171]: 153, 143, 135, 127, 125, 123, 109, 99, 61 MS^2^ [655]: 637, 611, 610, 609, 608, 605, 595, 593, 592, 580, 517, 493	Trihydroxy-octadecadienoic acid Ligstroside derivative
**37**	72.95	407	MS^2^ [407]: 389, 363, 346, 317, 255, 241, 195 MS^3^ [407→389]: 371, 361, 345, 327, 319, 301, 285, 259, 243, 221, 161	10-Hydroxy-10-Methyl oleuropein aglycone
**38**	77.45	409	MS^2^ [409]: 391, 382, 349, 329, 315, 306, 289, 277, 261, 253, 225, 197, 185, 155, 129	Oleuropein aglycon derivative

## Data Availability

The original data presented in the study are openly available at https://drive.google.com/drive/folders/1xHzmTus-aXua5JSAJ33v2V1u6owctwMc?usp=drive_link, accessed on 8 September 2026.

## Abbreviations

The following abbreviations are used in this manuscript:

3,4-DHPEA	3,4-Dihydroxyphenylethanol (hydroxytyrosol)
CaP	Calcium phosphate
H_2_O_2_	Hydrogen peroxide
HT	Hydroxytyrosol
MAPK	Mitogen-activated protein kinase
NOX	NADPH oxidases
OC	Osteoclast
OOD	Olive oil dregs
OOD H_2_O	Aqueous OOD extract
OOD MeOH	Methanolic OOD extract
PC	Phenolic compound
RANKL	Receptor activator of nuclear factor kappa-B ligand
ROS	Reactive oxygen species
SEM	Scanning Electron Microscopy
Std	Standard
TRAP	Tartrate-resistant acid phosphatase
